# Safety and Efficacy of Liraglutide on Cardiovascular Outcomes in Patients With Diabetes Mellitus: A Meta-Analysis of Randomized Controlled Trials

**DOI:** 10.7759/cureus.45421

**Published:** 2023-09-17

**Authors:** Hema Srikanth Vemulapalli, Jaahnavi Vajje, Wajeeh Rehman, Ghazala S Virk, Krushi Shah, Sandipkumar S Chaudhari, Irfan-ud-din Mian, Faraz Saleem

**Affiliations:** 1 Cardiology, Mayo Clinic, Phoenix, USA; 2 Internal Medicine, Dr. Pinnamaneni Siddhartha Institute of Medical Sciences and Research Foundation, Vijayawada, IND; 3 Internal Medicine, United Health Services Hospitals, State University of New York Upstate Medical University Binghamton Campus, Johnson City, USA; 4 Internal Medicine, Avalon University School of Medicine, Ohio, USA; 5 Internal Medicine, Gujarat Medical Education and Research Society (GMERS) Medical College, Gandhinagar, IND; 6 General Physician, Lions General Hospital, Mehsana, IND; 7 General Practice, General Hospital, Vadnagar, IND; 8 Medicine, Combined Military Hospital (CMH) Lahore Medical College and Institute of Dentistry, Lahore, PAK; 9 Internal Medicine, California Institute of Behavioral Neurosciences and Psychology, Fairfield, USA; 10 Internal Medicine, Akhtar Saeed Medical and Dental College, Lahore, PAK

**Keywords:** randomized controlled trials, meta-analysis, diabetes mellitus, patients, cardiovascular outcomes, liraglutide, efficacy, safety

## Abstract

Diabetes mellitus (DM) is a chronic metabolic disorder, with type 2 diabetes (T2DM) significantly impacting the cardiovascular (CV) system. Our comprehensive study on the cardiovascular effects of liraglutide, conducted concurrently with the formulation of diabetes treatment guidelines, aims to provide healthcare providers and patients with reassurance regarding the safety and effectiveness of liraglutide.

From the beginning until August 20, 2023, we conducted searches in databases including PubMed, Web of Science, Embase, Cochrane Library, Scopus, and Google Scholar. These searches aimed to identify studies comparing liraglutide to control in terms of symptom resolution among patients with T2DM. For all relevant outcomes, we calculated risk ratios along with their corresponding 95% confidence intervals.

Thirteen randomized controlled trials (RCTs) were included in this analysis. The results demonstrated a significant reduction in the risk of major adverse cardiovascular events (MACE), myocardial infarction, CV mortality, and all-cause mortality. No significant difference was found between the liraglutide and control groups for the outcome of stroke. However, sensitivity analysis revealed a significant reduction in the risk of stroke among patients taking liraglutide. Our comprehensive meta-analysis strongly supports the use of liraglutide for managing cardiovascular disease (CVD) due to its established safety and effectiveness. Further RCTs and meta-analyses are needed to more thoroughly evaluate liraglutide's therapeutic potential, with the aim of enhancing the quality of life for those with CVD.

## Introduction and background

Diabetes mellitus (DM), as defined by the World Health Organization (WHO), is a chronic metabolic disorder. It is characterized by elevated levels of blood glucose, gradually leading to detrimental effects on various organs such as the heart, eyes, vasculature, nerves, and kidneys. More than 90% of DM cases are classified as type 2 diabetes mellitus (T2DM), characterized by insufficient insulin production by pancreatic β-cells, inadequate insulin secretion, and tissue resistance to insulin [1,2]. The prevalence of diabetes in individuals aged ≥20 years is 9.5%, resulting in 21.8 million diagnosed cases among noninstitutionalized United States (U.S.) civilians [3].

At the clinical level, T2DM is significantly correlated with consequences affecting both the macrovascular and microvascular systems. These encompass nephropathy, retinopathy, neuropathy, peripheral arterial disease (PAD), cerebrovascular disease, and ischemic heart disease (IHD) [4]. DM triples cardiovascular (CV) mortality risk and doubles total mortality risk [5,6], but concerns over the potential CV effects of certain glucose-lowering treatments remain to be definitively confirmed [7,8]. Thus, the U.S. Food and Drug Administration (FDA) revised industry rules to mandate CV risk assessment for new diabetic drugs through rigorous randomized controlled trials (RCTs) [7,8]. Concentrating on the complex diabetes-cardiovascular mortality relationship is essential due to increased mortality risk. Our investigation aims to uncover therapeutic interventions targeting mortality-contributing factors [7,8].

Glucagon-like peptide 1 (GLP-1) and glucose-dependent insulinotropic polypeptide (GIP) are endogenous hormones responsible for the incretin effect [5,8,9]. GLP-1 is crucial for beta cell formation, inducing insulin production, and enhancing insulin gene expression, thus significantly reducing blood glucose levels and modifying T2DM's course and progression [5,8,9]. GLP-1-based medications' glucose-lowering and weight-reducing benefits impact CV factors, with recent research suggesting additional CV benefits unrelated to glucose reduction. Experimental models demonstrate diverse CV effects of GLP-1, including heart rate modulation, vascular tone, myocardial contractility, blood pressure regulation, and protection against ischemia-reperfusion injury [9].

Liraglutide, an extended version of GLP-1, shares a 97% amino acid sequence similarity with human GLP-1. Scientific evidence supports Liraglutide's efficacy in reducing glucose levels, as evidenced by trials showing slight declines in blood pressure and body weight [10-12]. The surge in liraglutide CV trials for type 2 diabetes prompted our FDA-protocol-aligned meta-analysis. We conclude that targeted diabetic therapy will revolutionize care. Our comprehensive CV effect study of liraglutide, aligned with developing diabetes treatment guidelines, aims to reassure healthcare providers and patients. This investigation aims to determine liraglutide's potential influence on major CV events in T2DM patients, underscoring its critical role as an intervention [10-12].

## Review

Methods

The current meta-analysis and systematic review adhere to a predetermined protocol in line with the Preferred Reporting Items for Systematic Reviews and Meta-Analyses (PRISMA) guidelines. Our analysis is based on previously documented investigations, so ethical approval and participant consent were deemed unnecessary [13].

Search Strategy and Selection Criteria

We conducted a comprehensive systematic search of Google Scholar, the Cochrane Library, PubMed, the Web of Science, Embase, and Scopus from their inception until August 20, 2023. The study keywords included "type 2 diabetes," "liraglutide," and "cardiovascular," along with the Boolean operators AND and OR. Detailed search strategy information is available in Appendix A.

The literature search, data extraction, and quality assessment were independently performed by two writers. Additional searches were conducted through references cited in the included studies to ensure comprehensive coverage. In cases where original research lacked important data, authors were contacted for clarification. Two authors independently screened and assessed study eligibility. Inclusion criteria were: (1) RCTs investigating liraglutide's efficacy and safety on cardiovascular outcomes in T2DM patients with and without pre-existing cardiovascular disease (CVD); (2) adult T2DM patients with > 12 weeks of disease duration and 7.0% glycated hemoglobin. The analysis involved comparing liraglutide-treated patients to those receiving alternative anti-diabetic treatment or a placebo. Studies with redundant/insufficient data for effect estimation and those involving non-diabetic or type 1 diabetic patients were excluded.

Data Extraction and Outcomes

Data extraction, conducted independently by two investigators, ensured the result's credibility. Disagreements were resolved through consensus, and a third reviewer was consulted if further clarification was needed. Data from studies meeting inclusion criteria were collected using a standard method. The information included the primary author's name, publication year, research country, intervention details, event and control quantities, baseline patient characteristics (age, gender, race, diabetes duration, HbA1c levels, body mass index), and occurrence of cardiovascular events.

The primary outcome assessed liraglutide's impact compared to alternative antidiabetic treatments or placebo on major adverse cardiovascular events (MACE). These events included nonfatal acute myocardial infarction (AMI), cardiovascular death, stroke, acute coronary syndromes, heart failure, or both. Secondary outcomes included myocardial infarction, stroke, all-cause mortality, and CV-related mortality.

Quality Assessment and Statistical Analysis

The quality assessment of clinical trials was independently conducted by two researchers using the Risk of Bias Tool 2 (ROB 2.0) (Appendix B1) [14]. This tool is widely employed for clinical trial quality evaluation. Statistical analysis and forest plot generation were performed using Review Manager 5.4. Forest plots using a random effects model determined the pooled effect size. A funnel plot assessed publication bias (Appendix B2). A p-value of <0.05 was considered statistically significant.

Results

Literature Search Results

**Figure 1:**
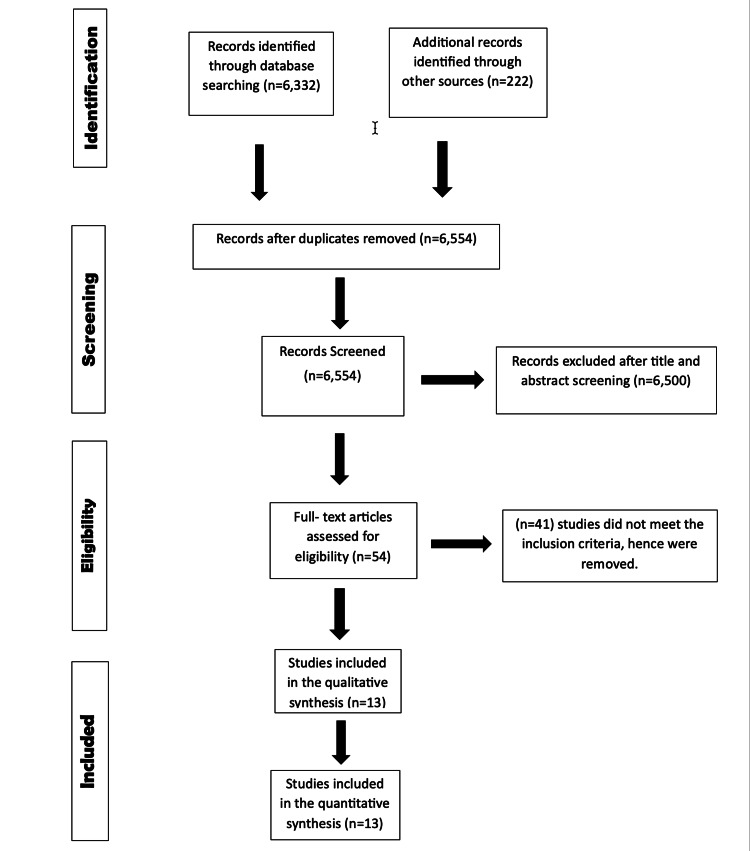
PRISMA flow diagram

The comprehensive screening process is depicted in Figure 1 through the PRISMA flow chart. After duplicate removal and filtering, 13 papers were evaluated.

Study Characteristics

Table 1 outlines study characteristics and initial participant demographics. The study included 50,751 participants with an average age of 59.4 years. Participants' mean BMI was 31.4, and all included patients had HbA1c levels exceeding 8.

**Table 1:** Baseline characteristics of the included studies

					Mean			
Author (year)	Study description	Liragitude	Comparators	Sex (M/F)	Age (years)	BMI (kg/m^2^)	Diabetes duration	HbA1c (%)
Marre et al. [15]	Add-on to glimepiride	695	114 (placebo)	405/404	56	30	7	8.4
Nauck et al. [16]	Add-on to metformin	724	232 (rosiglitazone) 121 (placebo)	462/465 495/350	56 57	29.8 31.2	7 7	8.4 8.4
Russell-Jones et al. [17]	Add-on to metformin and glimepiride	232	242 (glimepiride) 115 (placebo)	560/406 189/158	57 57	31.2 30.3	7 9	8.4 8.3
Zinman et al. [18]	Add-on to metformin and rosiglitazone	355	234 (glargine) 175 (placebo)	273/193 302/228	57 55	30.8 33.7	9 9	8.3 8.5
Pratley et al. [19]	Monotherapy	446	219 (Sitagliptin)	352/313	55	32.8	6	8.4
Seino et al. [20]	Monotherapy	268	132 (Glibenclamide)	268/132	58	24.4	8	8.8
Garber et al. [21]	Monotherapy	498	248 (glimepiride)	371/375	53	33	5	8.3
Marso et al. [22]	Monotherapy	4668	4672 (placebo)	6003/3337	64.3	32.5	12.7	8.7
Verma et al. [23]	Monotherapy	4668	4672 (placebo)	6003/3337	64.4	32.6	12.8	8.7
Mann et al. [24]	Monotherapy	1116	1042 (placebo)	1322/836	67.3	32.7	15.2	8.7
Nauck et al. [25]	Monotherapy	4668	4672 (placebo)	6003/3337	64.3	n/a	12.9	8.7
Gilbert et al. [26]	Monotherapy	3053	3130 (placebo)	3935/2248	65.8	32.4	13.2	8.7
Marso et al. [27]	Monotherapy	4668	4672 (placebo)	6003/3337	64.2	33.1	12.4	8.7

Results of Meta-Analysis

**Figure 2:**
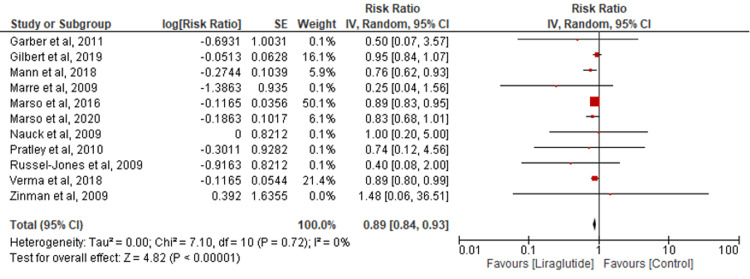
Comparison of MACE MACE: major cardiovascular events [15-19,21-27]

MACE: Pooling results from 11 studies assessed MACE outcomes. Liraglutide significantly reduced MACE risk compared to the control group (RR=0.89; 95% CI: 0.84-0.93; P<0.00001; I2=0%) (Figure 2).

**Figure 3:**
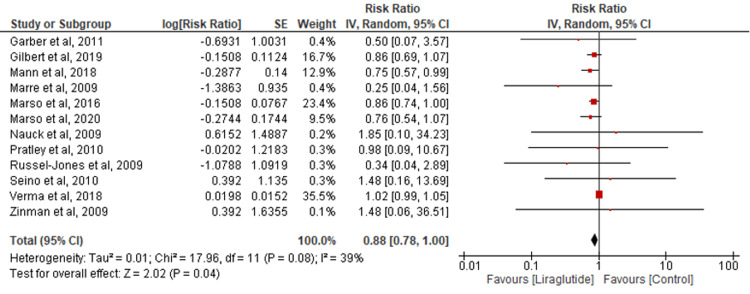
Comparison of myocardial infarction [15,17-27]

Myocardial infarction: Twelve studies were combined to assess myocardial infarction occurrence. Liraglutide significantly reduced myocardial infarction incidence compared to the control group (RR=0.88; 95% CI: 0.78-1.00; P=0.04; I2=39%) (Figure 3). Sensitivity analysis confirmed consistent results with reduced heterogeneity (RR=0.83; 95% CI: 0.74-0.92; P=0.0005; I2=0%) (Appendix C).

**Figure 4:**
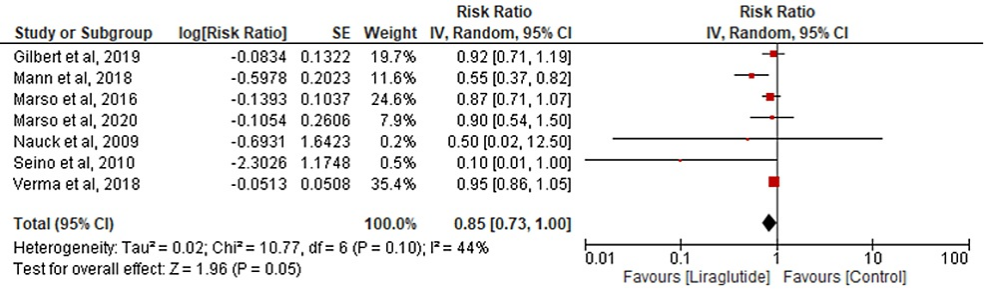
Comparison of stroke [16,20,22-24,26-27]

Stroke: Six studies evaluated stroke outcomes. No significant difference between liraglutide and control groups was found (RR=0.85; 95% CI: 0.73-1.00; P=0.05; I2=44%) (Figure 4). Sensitivity analysis indicated a significantly lower stroke incidence in the liraglutide group with reduced heterogeneity (RR=0.82; 95% CI: 0.69-0.98; P=0.03; I2=22%) (Appendix D).

**Figure 5:**
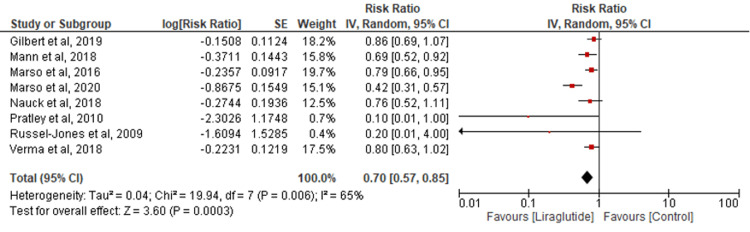
Comparison of cardiovascular disease death [17,19,22-27]

CVD death: Eight studies were pooled to assess CVD death. Liraglutide significantly reduced CVD death incidence compared to the control group (RR=0.70; 95% CI: 0.57-0.85; P=0.0003; I2=65%) (Figure 5). Sensitivity analysis maintained consistent results with significantly reduced heterogeneity (RR=0.79; 95% CI: 0.71-0.88; P<0.0001; I2=0%) (Appendix E).

**Figure 6:**
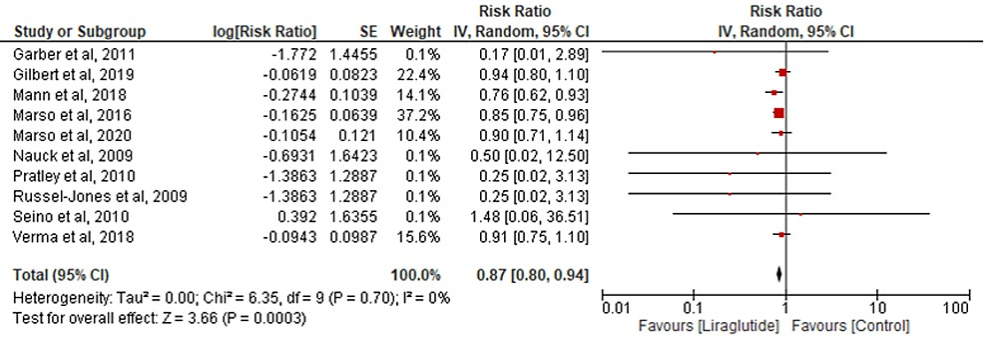
Comparison of all-cause death [16-17,19,20-24,26-27]

All-cause death: Data from ten studies were aggregated to assess all-cause death. Liraglutide significantly reduced all-cause death incidence compared to the control group (RR=0.87; 95% CI: 0.80-0.94; P=0.0003; I2=0%) (Figure 6).

Discussion

This meta-analysis aimed to assess the impact of liraglutide on CV outcomes in patients with type 2 diabetes mellitus (T2DM) with or without baseline CVD. Liraglutide demonstrated improved outcomes in MACE, myocardial infarction, CVD mortality, and all-cause mortality when compared to the control group. The two groups did not exhibit any statistically significant difference in terms of stroke outcomes. However, upon sensitivity analysis, liraglutide was associated with a decreased risk of stroke compared to the control group.

Addressing metabolic risk factors has the potential to reduce both morbidity and mortality rates in individuals with T2DM. The efficacy of GLP-1 receptor agonists (GLP-1 RA) in lowering blood pressure, HbA1c levels, and other metabolic risk markers has been established in multiple studies [28-31]. GLP-1 activates adenylate cyclase through the GLP-1 receptor (GLP-1R) and the stimulatory Gs protein, indicating its potential cardio-protective and vasodilatory actions, which might not be receptor-dependent [32-34]. Additionally, GLP-1's role in this phenomenon is plausible. The primary GLP-1 metabolite has elevated circulation levels compared to intact bioactive GLP-1. Notably, CVD and all-cause mortality have been associated with HbA1c [34- 36].

The meta-analysis of trials such as United Kingdom Prospective Diabetes Study (UKPDS), PROactive, ADVANCE, VADT, and Action to Control Cardiovascular Risk in Diabetes (ACCORD) provides evidence for the moderate advantages of glucose management in T2DM patients, as demonstrated by diverse outcomes [37]. Liraglutide treatment's efficacy in reducing CV risk biomarkers, like TNF-α and MR-proADM, has been proven in a randomized, placebo-controlled, double-blind crossover study [38]. The available data compellingly indicate that pharmacological interventions targeting HbA1c levels significantly impact CV outcomes beyond HbA1c reduction alone. Recent clinical research suggested that albiglutide, a GLP-1 RA, did not reduce MACE risk in T2DM patients [39]. Potential explanations for this discrepancy could involve the statistical power of our investigation and the influence of GLP-1 receptor agonists on heart rate, which has been linked to an increased risk of cardiovascular complications [40,41].

Several prior meta-analyses have assessed liraglutide's efficacy and safety on CV outcomes. Bethel et al. showed a significant decrease in MACE (HR 0.90, 95% CI 0.82-0.99; p=0.033) and all-cause mortality (0.88, 0.81-0.95; p=0.002), but did not find any effect of GLP-1 on myocardial infarction and stroke incidence [42]. Similarly, Giugliano et al. found reduced MACE (HR=0.86, 95% CI 0.79-0.94, P=0.006) and CV mortality, along with decreased stroke incidence, but no significant impact on myocardial infarction [43]. Qin and Song reported lower MACE and stroke incidence with GLP-1 treatment but no effect on myocardial infarction [44]. In contrast, the study by Duan et al. significantly lowered MACE, myocardial infarction, all-cause mortality, and CV mortality with liraglutide while failing to show an effect on stroke [45]. While mostly consistent, our study uniquely demonstrated a significant reduction in myocardial infarction and stroke incidence.

Beyond its immediate clinical implications, liraglutide's notable cardiovascular benefits herald a positive future for healthcare. Liraglutide's effectiveness in improving CV outcomes emphasizes the need to address metabolic diseases holistically, beyond traditional disease-specific boundaries. This discovery encourages researchers and pharmaceutical companies to explore novel therapeutic approaches that simultaneously target diabetes and cardiovascular conditions. Multi-target drugs addressing lipid profiles, CV function, and glucose metabolism interactions could revolutionize treatment approaches. Additionally, liraglutide's success may stimulate the development of personalized medicine tailored to individual metabolic and CV profiles.

Limitations

Our study is constrained by potential misdiagnosis due to varying incident CVD diagnostic criteria. In trials with smaller subgroup sizes and no prior CVD history, liraglutide's effects were less statistically significant. The high-risk focus on type 2 diabetes might limit generalizability to lower-risk populations, and medication variations could introduce bias. Some of the examined studies focused on liraglutide's impact on glycemic control and safety, indicating the need for larger, well-designed RCTs to comprehensively evaluate CV outcomes.

## Conclusions

The outcomes of our thorough investigation and meta-analysis strongly support the use of liraglutide for managing CVD outcomes, given its proven safety and effectiveness. Further RCTs and additional meta-analyses are warranted to further evaluate liraglutide's therapeutic potential with the objective of enhancing the quality of life for individuals affected by CVD.

## Figures and Tables

**Figure 7:**
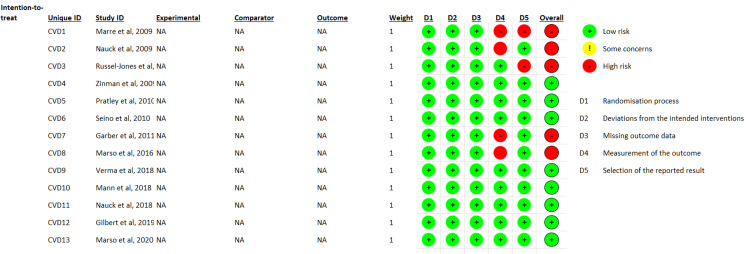
Quality assessment

**Figure 8:**
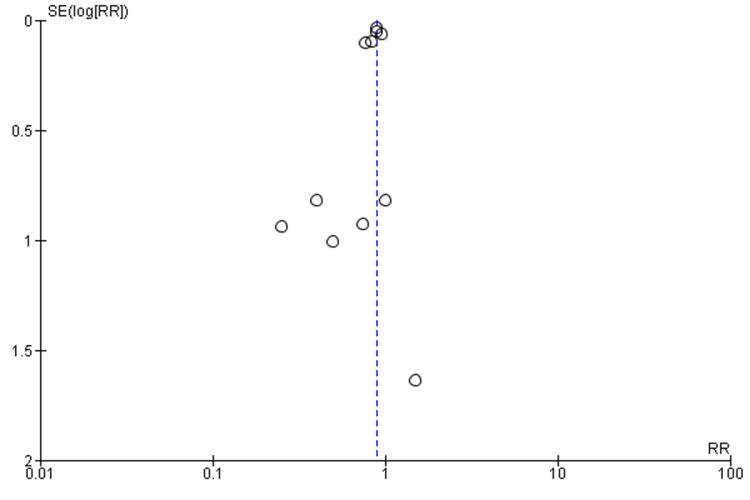
Funnel plot of comparison: 1 CVD outcomes, outcome: MACE

**Figure 9:**
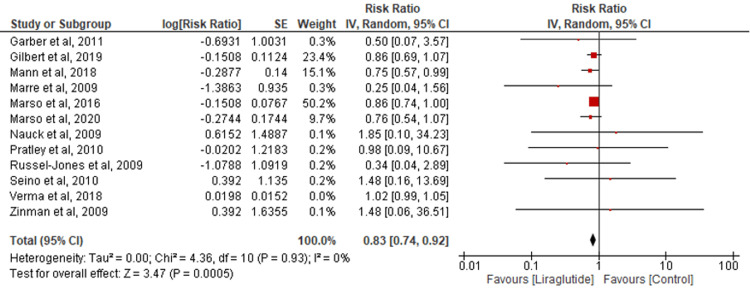
Sensitivity analysis of the outcome of myocardial infarction

**Figure 10:**
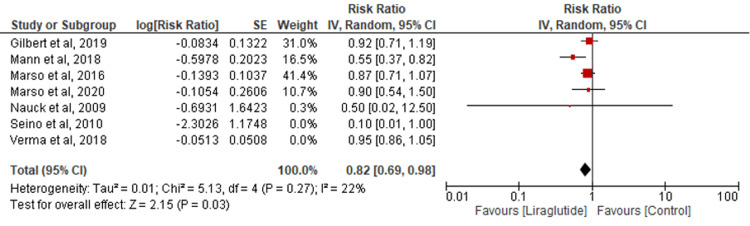
Sensitivity analysis of the outcome of stroke

**Figure 11:**
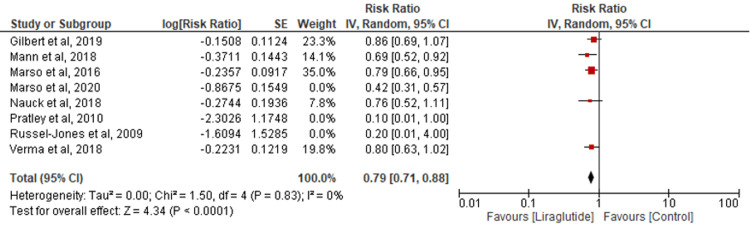
Sensitivity analysis of the outcome of CVD death

## Appendices

Appendix A

**Table 2:** Search strategy

Database	Query	Search details	Results
PubMed	((((Liraglutide) AND (Diabetes Mellitus)) OR (DM)) AND (cardiovascular disease))) AND (randomized controlled trial)	((("liraglutid"[All Fields] OR "liraglutide"[MeSH Terms] OR "liraglutide"[All Fields] OR "liraglutide s"[All Fields]) AND ("diabetes mellitus"[MeSH Terms] OR ("diabetes"[All Fields] AND "mellitus"[All Fields]) OR "diabetes mellitus"[All Fields])) OR ("dyn med"[Journal] OR "dis mon"[Journal] OR "dis manag"[Journal] OR "dm"[All Fields])) AND ("cardiovascular diseases"[MeSH Terms] OR ("cardiovascular"[All Fields] AND "diseases"[All Fields]) OR "cardiovascular diseases"[All Fields] OR ("cardiovascular"[All Fields] AND "disease"[All Fields]) OR "cardiovascular disease"[All Fields]) AND ("randomized controlled trial"[Publication Type] OR "randomized controlled trials as topic"[MeSH Terms] OR "randomized controlled trial"[All Fields] OR "randomised controlled trial"[All Fields])	1,110
Web of Science			
Embase			
Cochrane Library			
SCOPUS			
Google Scholar			

Appendix B1 and B2

Appendix C

Appendix D

Appendix E
